# Comparative in vitro and in vivo Evaluation of Different Iron Oxide-Based Contrast Agents to Promote Clinical Translation in Compliance with Patient Safety

**DOI:** 10.2147/IJN.S402320

**Published:** 2023-04-21

**Authors:** Harald Unterweger, Christina Janko, Tamara Folk, Iwona Cicha, Noémi Kovács, Gyula Gyebnár, Ildikó Horváth, Domokos Máthé, Kang H Zheng, Bram F Coolen, Erik Stroes, János Szebeni, Christoph Alexiou, László Dézsi, Stefan Lyer

**Affiliations:** 1 ENT-Department, Section of Experimental Oncology und Nanomedicine (SEON), Universitätsklinikum Erlangen, Erlangen, Germany; 2 Hungarian Centre of Excellence for Molecular Medicine, Semmelweis University, Budapest, Hungary; 3 Medical Imaging Centre, Semmelweis University, Budapest, Hungary; 4 Department Biophysics and Radiation Biology, Semmelweis University, Budapest, Hungary; 5 Department of Vascular Medicine, Amsterdam University Medical Centers, Amsterdam Cardiovascular Sciences, University of Amsterdam, Amsterdam, the Netherlands; 6 Department of Biomedical Engineering and Physics, Amsterdam University Medical Centers, Amsterdam Cardiovascular Sciences, University of Amsterdam, Amsterdam, the Netherlands; 7 Nanomedicine Research and Education Center, Institute of Translational Medicine, Semmelweis University, Budapest, Hungary; 8 SeroScience Ltd, Budapest, Hungary

**Keywords:** magnetic resonance imaging, MRI, nanomedicine, nanoparticles, complement activation, CARPA

## Abstract

**Introduction:**

One of the major challenges in the clinical translation of nanoparticles is the development of formulations combining favorable efficacy and optimal safety. In the past, iron oxide nanoparticles have been introduced as an alternative for gadolinium-containing contrast agents; however, candidates available at the time were not free from adverse effects.

**Methods:**

Following the development of a potent iron oxide-based contrast agent SPION^Dex^, we now performed a systematic comparison of this formulation with the conventional contrast agent ferucarbotran and with ferumoxytol, taking into consideration their physicochemical characteristics, bio- and hemocompatibility in vitro and in vivo, as well as their liver imaging properties in rats.

**Results:**

The results demonstrated superior in vitro cyto-, hemo- and immunocompatibility of SPION^Dex^ in comparison to the other two formulations. Intravenous administration of ferucarbotran or ferumoxytol induced strong complement activation-related pseudoallergy in pigs. In contrast, SPION^Dex^ did not elicit any hypersensitivity reactions in the experimental animals. In a rat model, comparable liver imaging properties, but a faster clearance was demonstrated for SPION^Dex^.

**Conclusion:**

The results indicate that SPION^Dex^ possess an exceptional safety compared to the other two formulations, making them a promising candidate for further clinical translation.

## Introduction

Imaging the structure and function of tissues and organs is often the key to reliable medical diagnosis and a prerequisite for effective treatment. One of the most important imaging modalities in this regard is magnetic resonance imaging (MRI) which utilizes magnetic fields to excite protons, sparing the patients the exposure to ionizing radiation. It shows good local resolution and soft tissue contrast already in its native form. Nevertheless, the use of MRI contrast agents is often required for early detection of various diseases by strongly enhancing signal contrast between healthy and non-healthy tissue. Despite the widespread use of gadolinium-based contrast agents (GBCAs), the current gold standard, they produce side effects, which include allergic and adverse reactions, but can also lead to nephrogenic systemic fibrosis in patients with renal failure.^1^,^2^ In 2014, Kanda et al were the first to report the deposition of GBCAs in the deep nuclei of the brain^3^ and multiple studies confirmed their findings afterwards.^4^,^5^ There is also evidence that GBCA deposition can occur in children.^6^ In a detailed review from 2017, Gulani et al summarized various studies on this topic and compared different gadolinium-based compounds.^7^ Their findings suggest that linear GBCAs show a higher retention capacity in the brain compared to macrocyclic GBCAs; however, the current data are not sufficient to claim whether a clinical implication is necessarily associated with this deposition. As a result of these developments, both the US Food & Drug Administration (FDA)^8^ and the European Medicines Agency (EMA)^9^ have issued warnings against the use of GBCAs and recommended suspension of some linear agents/formulations. While the exact relationships between gadolinium deposition in the body and clinical implications are the subject of current investigations, alternatives to GBCAs are being sought and explored. In this context, superparamagnetic iron oxide nanoparticles (SPIONs) currently represent an interesting alternative contrast agent. Having strong magnetic susceptibility effects resulting in increased T_2_ and T_2_* related signal attenuation, they usually produce negative contrast with respect to healthy tissue, but under specific conditions positive/bright contrast is also feasible.^10^ The strong signal decay associated with the presence of SPIONs makes them detectable in relatively low concentrations and therefore allow an increase in MRI sensitivity almost to the cellular level, as well as enable in vivo cell visualization and tracking.^11–13^ Previously, only two SPION-based intravenous agents were clinically approved, namely ferumoxide (trade names: Feridex^®^, Endorem^®^) and ferucarbotran (trade name: Resovist^®^), but both were withdrawn from the market, mainly due to economic reasons. Nevertheless, Resovist^®^, available in Japan, is still commonly used as benchmark for new imaging techniques and devices.^14–18^ Currently, however, there is a growing interest in the use of ferumoxytol (trade names: Rienso^®^, Feraheme^®^) as a SPION-based contrast agent for MRI. Ferumoxytol is indicated only as an intravenous iron replacement drug in adult patients with iron deficiency anemia, in particular those with chronic kidney disease or renal failure,^19^,^20^ but is being used off-label for MRI.^21–23^ The number of clinical trials using ferumoxytol either as a replacement for GBCAs or for completely new imaging modalities is steadily increasing. For example, in a retrospective study, ferumoxytol was shown to provide an equivalent assessment of the extent of bone and soft tissue sarcomas compared with GBCA.^24^ It was also shown that ferumoxytol is equivalent to GBCA for the detection of intracranial metastases and could be a suitable alternative, especially when GBCAs are contraindicated.^25^ Other example applications of ferumoxytol in MRI include the visualization of neurological features,^26^,^27^ of acute kidney allograft rejection in pediatric patients,^28^ or of myocardial hypoperfusion.^28^

Despite the promising clinical results, there are studies showing that SPION-based contrast agents can lead to considerable side effects. For example, administration of ferumoxide or ferucarbotran was associated with adverse events in 10% and 7.1% of all cases, respectively.^29^ For ferucarbotran, there is also evidence that it can negatively affect autoimmune-mediated neuroinflammation after a single exposure.^30^ A multicenter study carried out between 2003 and 2018 showed that ferumoxytol also tends to cause mostly mild side effects in 1.8% and moderate side effects in 0.2% of the patients, including hypertension, nausea, backache, headache, and vomiting.^31^ However, cases of acute hypersensitivity were also reported. For this reason, ferumoxytol received the FDA’s strongest type of warning in 2015, due to serious risk of potentially fatal anaphylactic reactions upon administration. The use of ferumoxytol for imaging may therefore pose a considerable risk to the patients.^32^ Altogether, this calls for a SPION-based system with even better biocompatibility and a more comfortable diagnostic regimen for the patient. In our previous studies, we developed a nanoparticulate system named SPION^Dex^, a type of SPIONs coated with crosslinked dextran, that fulfills these requirements.^33^,^34^ These particles can be easily and reproducibly tuned in size and be stored for several months without any sign of agglomeration or sedimentation.^34^ In vitro and in vivo results have also indicated that SPION^Dex^ represent an extremely biocompatible and non-immunogenic system, yet their direct comparison with ferucarbotran and ferumoxytol has not been reported so far. The current study thus aims at a systematic in vitro and in vivo comparison between the iron oxide-based nanosystems that can serve as MRI contrast agents in terms of biocompatibility and imaging performance, in order to evaluate their risk-to-benefit profile and the potential advantages of SPION^Dex^ in the clinical application.

## Materials and Methods

### Preparation of Nanoparticulate Contrast Agents

SPION^Dex^ were produced according to a modified protocol that was previously published.^33^ In short, ferric and ferrous chloride salts were dissolved in a 10% (w/w) aqueous dextran solution at a molar Fe^3+^:Fe^2+^ ratio of 2:1. The solution was cooled to 4 °C and dextran-coated iron oxide particles were precipitated with ammonia. After heating to 75 °C and subsequent cooling to room temperature, excess educts and byproducts were removed via tangential flow filtration (TFF; Repligen, USA) using a hollow fiber PES membrane (cut-off: 100 kDa). Next, sodium hydroxide was added to the particles and the dextran shell was crosslinked with epichlorohydrin. Eventually, the product was again washed via TFF, sterile filtered (0.2 µm) and stored at 4 °C until further use. All synthesis reagents were purchased from Merck, Germany, and are of Ph Eur purity were available, otherwise of *purum* quality. Resovist^®^ was obtained from Schering Deutschland GmbH (Berlin, Germany) and Feraheme^®^ from AMAG Pharmaceuticals (Waltham, USA).

### Physicochemical Characterization of Nanoparticles

The iron concentration of the particles was determined via microwave plasma atomic emission spectroscopy (MP-AES; MP-4200 from Agilent Technologies, USA), using a commercially available iron standard solution (Bernd Kraft, Germany). The hydrodynamic size distributions, polydispersity indices and ζ-potentials at pH 7.4 were determined with a Malvern Nano ZS (United Kingdom) in backscattering mode. Thermogravimetric analysis was performed with freeze-dried nanoparticles using a TG 209 Libra (Netzsch, Germany). Fourier transform infrared spectroscopy (FTIR, ALPHA from Bruker, USA) was used to compare the different particle coatings. The magnetic behavior was investigated by measuring the magnetic volume susceptibility with a MS3/MS2G susceptometer (Bartington, United Kingdom). The MRI properties of the different iron oxide-based contrast agents were investigated with a 3 T Philips Ingenia MRI system (Netherlands). Thereby, T_1_, T_2_ and T_2_* maps were scanned at different iron concentrations c_np_ and the images were processed using the VivoQuant 1.22 pre-clinical software (USA). The respective relaxivities r_i_ were derived from the relaxation rates R_i_ using the following equation^35^:

(1) $${{\rm{R}}_{\rm{i}}}{\rm{ = }}{{\rm{1}} \over {{{\rm{T}}_{\rm{i}}}}}{\rm{ = }}{{\rm{r}}_{\rm{i}}}{\rm{ \times }}{{\rm{c}}_{{\rm{np}}}}{\rm{ + }}{{\rm{R}}_{\rm{d}}}$$

where i reflects the scan mode (1 for T_1_ and so on), T_i_ stands for the relaxation time and R_d_ is the diamagnetic relaxation rate of the dispersion medium.

Three independent experiments were performed with triplicate samples, and the results were averaged.

### Examination of in vitro Cytocompatibility

The non-adherent, human T-cell leukemia cell line Jurkat (ACC 282, German Collection of Microorganisms and Cell Cultures GmbH, Germany) was used for the examination of the cytocompatibility. The cells were cultivated with RPMI 1640 medium supplemented with 10 vol% fetal bovine serum (FBS) and 1 vol% glutamine, in a cell culture incubator (INCOmed, Memmert, Germany) at 37 °C, 95% humidified air, and 5% CO_2_. 2.5 × 10^5^ Jurkat cells were seeded into each well of a 48 well plate and treated with different iron oxide-based contrast agents at a concentration range between 25 and 400 µg Fe/mL. Water served as negative control and 2% DMSO served as positive control. Every sample was prepared in triplicate. After incubation for 24 and 48 h, aliquots were stained with a freshly prepared staining solution containing AnnexinA5-FITC (AxV), Hoechst 33342 (Hoe), Hexamethylindodicarbo-cyanine iodide dye (DiI), and propidium iodide (PI).^36^ In addition, the cell cycle and DNA degradation were investigated using propidium iodide staining in the presence of Triton X-100 (PIT).^37^ In both cases, cell staining was examined via flow cytometry using a Gallios cytofluorometer (Beckman Coulter, USA). AxV is excited at 488 nm and recorded at 525/38 nm band pass (BP), PI is excited at 488 nm as well, but recorded at 620/30 nm BP, and Hoe is excited at 405 nm and recorded at 430/40 nm BP. Electronic compensation was used in order to eliminate any fluorescence bleed-through. Similarly, potential occurrence of iron induced reactive oxygen species (ROS) and the level of reduced glutathione were investigated using the fluorescent dyes 2’-7ʹdichlorofluorescin (DCFH) and monobromobimane (MBB), respectively.^38^ Here, cells treated with 1 mM H_2_O_2_ served as positive control. Flow cytometry was performed to measure DCFH and MBB fluorescence after 24 h of incubation with respective doses of contrast agents. In general, flow cytometry data were analyzed with Kaluza software 1.2 from Beckman Coulter (USA) and processed using Microsoft Excel (USA). All experiments were performed in triplicate, five times independently; the results were averaged.

### In vitro Hemo- and Immunocompatibility

For hemocompatibility testing, blood was drawn from healthy volunteers. The use of human material was approved by the local ethics committee at the University Hospital Erlangen (# 257 14B) and the donors provided informed consent, in accordance with the Declaration of Helsinki. Colloidal stability of the nanoparticulate formulations in blood from three healthy volunteer donors was tested in the presence of three different anticoagulants, ie, lithium-heparin, ethylenediaminetetraacetic acid (EDTA), and sodium citrate. 30 min after incubation, blood smears were prepared on glass slides and examined for nanoparticle agglomerates (>200 nm) by transmission light microscopy.

Hemolytic properties: The hemoglobin content of lithium-heparin-anticoagulated whole blood was adjusted to 5 mg/mL in phosphate buffered saline (PBS). Nanoparticles were diluted in H_2_O and incubated with the diluted blood for 3 h at 37 °C (ratio 1:10) and carefully mixed every 30 min. One percent Triton X-100 served as positive control, PBS served as negative control. H_2_O was tested as vehicle control. To detect potential interferences of nanoparticles with the assay, the positive control was additionally spiked with nanoparticles. To determine the nanoparticle background, nanoparticles diluted in H_2_O at the respective concentrations were used. After 3 h, samples were centrifuged at 800× *g* for 15 min. The supernatant was centrifuged again for 4 h at 18,000× *g* to sediment the nanoparticles. In order to determine the content of free hemoglobin, 100 μL supernatant was incubated with 100 μL Drabkin’s reagent (Sigma-Aldrich) for 5 min in 96-well plates at 60 °C while heating. Drabkin’s reagent lyses erythrocytes and converts hemoglobin and its derivatives to methemoglobin and then to cyanmethemoglobin. Absorption of the samples was measured at 590 nm on Microplate Reader Filter Max F5 (Molecular Devices, USA).

Plasma coagulation: Sodium citrate anticoagulated blood was centrifuged at 2500× g for 15 min to obtain platelet poor plasma (PPP). Nanoparticles were diluted in H_2_O and incubated with PPP for 30 min at 37 °C (ratio 1:10). Thrombin time (TT), prothrombin time (PTT), and activated partial thromboplastin time (aPTT) were determined using coagulation kits obtained from DiaSys Diagnostic Systems GmbH (Holzheim, Germany) according to the manufacturer’s instructions. The experiments were performed in the MC 4 Plus coagulometer (Merlin medical, Germany). Plasma samples with normal and abnormal coagulation times (obtained from DiaSys Diagnostic Systems GmbH, Germany) were used as negative and positive controls, respectively.

Complement activation: To analyze complement activation, an iC3b ELISA obtained from MicroVue (Quidel, USA) was performed. For this, sodium citrate anticoagulated blood was centrifuged for 10 min at 2500× g to obtain platelet-poor plasma (PPP). The PPP was mixed 1:1 with Veronal Buffer (Lonza, Switzerland). All nanoparticles were diluted to 1.2 and 0.3 mg/mL in H_2_O and possessed an endotoxin level <0.1 EU/mL. Twenty-five microliters of the nanoparticles were added to 50 µL of the mixture of PPP and Veronal Buffer. H_2_O served as negative control, cobra venom factor (CVF) and cremophor (both Quidel, USA) served as positive controls for complement activation. Additional samples with nanoparticles and CVF were prepared to analyze possible interferences by the nanoparticles (inhibition/enhancement controls, IEC). All samples were incubated at 37 °C for 30 min. Then, the samples were diluted in Specimen Diluent (CFV and IEC 1:25, other samples 1:100). The ELISA was performed according to the manufacturer’s instructions. In brief, 100 µL iC3b Specimen Diluent (blank), standards, controls and samples were added to the assay plate and incubated for 30 min at RT. Then, the plate was washed five times with the washing buffer, followed by incubation with 50 µL iC3b conjugate for 30 min at RT. After that, the plate was again washed five times. Next, 50 µL 1× substrate solution was added and incubation continued for 30 min. Subsequently, 50 mL stop solution was added and the absorbance at 405 nm was analyzed with the Microplate Reader Filter Max F5 (Molecular Devices, USA). Thrombocyte activation: Platelet-rich plasma (PRP) was obtained from sodium citrate anticoagulated blood (centrifugation at 200× g for 30 min). PBS served as negative, H_2_O as vehicle, and collagen (Collagen I, Rat Tail, Gibco, Life Technologies Corporation, USA) as positive control, respectively. The nanoparticles were diluted to 4 mg/mL and 1 mg/mL. Ninety microliter PRP was mixed with 10 µL sample into a prewarmed reaction tube and incubated for 15 min at 37 °C with continuous shaking. Subsequently, 50 µL of the samples were added to a FACS tube containing 100 µL PBS and the number of non-aggregated platelets was determined by flow cytometry. Formation of neutrophil extracellular traps (NETosis): Polymorphonuclear leukocytes (PMN) were isolated from whole blood using ficoll density gradient centrifugation (Merck, Germany). PMN were adjusted to a density of 1 × 10^6^ cells/mL and 225 µL was added to cell culture plates. Nanoparticles were diluted to 0.5, 1, 2, and 4 mg/mL. Phorbol-12-myristate-13-acetate (PMA) at 1 mM served as positive control. Cell medium and H_2_O served as negative and vehicle controls. Twenty-five microliters of each sample was added to the cells and incubated for 4 h. Free PMN as well as agglomerates were analyzed in flow cytometry. In general, flow cytometry data were analyzed with Kaluza software 1.2 from Beckman Coulter (USA) and processed using Microsoft Excel (USA). All experiments were performed in triplicate, three times independently and the results were averaged.

### In vivo Complement Activation in a Porcine Model

The protocol of Szebeni et al^39^,^40^ was used to study the activation of the complement system in vivo. Briefly, domestic male Yorkshire pigs with a body weight of 20–25 kg were prepared for the treatment with different contrast agents by intubation with endotracheal tubes and anesthetization with isoflurane (2–3 vol%) in oxygen. All test substances were injected into the animals as a bolus via the left external jugular vein. The used contrast agent doses were 0.5 and 5.0 mg Fe/kg. Saline served as negative control and zymosan at a concentration of 0.1 mg/mL served as positive control. During the procedure, a CAP10 capnograph (Medlab, Germany) monitored the respiratory rate, as well as the end-expiratory CO_2_ concentration, and the temperature was measured rectally. A pulse oximeter was fixed on the tail and the pulmonary artery pressure (PAP) was acquired through a Swan-Ganz catheter (AI-07124; Teleflex, Ireland) which was introduced into the pulmonary artery via the right jugular vein. The systemic arterial pressure (SAP) was recorded in the femoral artery. These hemodynamic changes were continuously monitored (sampling rate: 1 kHz) with an AD Instruments PowerLab System (Bella Vista, Australia) using LabChart Pro v6 software. In order to determine changes in platelets, white blood cells (WBC) and thromboxane B2 (TXB2) levels in plasma with an enzyme-linked immunosorbent assay (ELISA; Cayman Chemical Co., USA), blood samples were collected before and after the administration of the CA. Experiments were performed in duplicate for each contrast agent and each concentration; the results were averaged. The study was approved by the local ethics committee for animal experimentation (ÁTET, Hungary, Permit No. PE/EA/353-7/2020). The guidelines for animal welfare and animal testing of the Government of Hungary were followed in this study (Decree No 40/2013 [II. 14.]).

### In vivo Magnetic Resonance Imaging in Rats

In vivo MRI studies were carried out with healthy male and female (50%/50%) Wistar rats (Janvier Labs, France) using a 3 T Philips Ingenia MRI system (Netherlands). A Philips 8-channel knee coil was used for the MRI acquisition and medetomidine/ketamine (0.5 mg/50 mg per kg body weight, respectively) was administered as intraperitoneal injection for animal anesthesia. The different formulations were administered via intravenous injection at a dose of 2.6 mg iron per kg body weight (mg Fe/kg), and animals were observed for signs of acute and delayed toxicity, afterwards. 2D multislice T_1-_weighted turbo spin-echo (T_1_w TSE), 2D multislice T_2_-weighted turbo spin-echo with spectral attenuated inversion recovery for fat suppression (T_2_w TSE SPAIR) and 3D 4-echo gradient echo T_2_*-weighted (T_2_*w FFE) imaging of the liver were performed before, as well as at 1, 3, 24, and 168 h after administration with the following parameters listed in Table 1. Imaging sequence parameters were chosen to minimize motion artifacts, which is why no respiratory gating was performed. Image analysis was made by manual region of interest (ROI) selection with the VivoQuant v. 1.22 software (USA). Hereby, the liver mean signal was normed to muscle mean signal on each scan to make the different scans comparable. The experiment was performed in triplicate for each formulation independently, and the results were averaged. The study was approved by the local ethics committee for animal experimentation (ÁTET, Hungary). The guidelines for animal welfare and animal testing of the Government of Hungary were followed in this study (Decree No 40/2013 [II. 14.]).

**Table 1:** Summary of MRI Acquisition Parameters for in vivo Imaging

Type of Scan	Repetition Time/Echo Time (ms/ms)	vField of View (mm³)	Resolution (mm³)	Gap Between Slices (mm)	Number of Animals
T_1_w TSE	630.8/20	70 × 70 × 57	0.3125 × 0.3125 × 1.5	0.8	3
T_2_w TSE SPAIR	2000/40	70 × 70 × 54	0.3125 × 0.3125 × 1.5	0.15	3
T_2_*w FFE	11.173/6.638	70 × 70 × 64.5	0.49 × 0.49 × 1.5	–	3

**Abbreviations**: T_i_w (i=1, 2 or 2*), T_i_-weighted, TSE, turbo spin echo, SPAIR, spectral attenuated inversion recovery, FFE, fast field echo.

### Statistical Analysis

Data are expressed as mean ± standard deviation, unless stated otherwise. Normality of the data was tested with the D’Agostino–Pearson omnibus K2 Test using Prism v. 9.4.1 (GraphPad Software, USA). After passed normality test, one-way analysis of variance (ANOVA) with the Bonferroni post-hoc test was performed in Prism in order to determine differences between the untreated control and the samples exposed to different contrast agent concentrations. Asterisks mark statistical significance: *p < 0.05, **p < 0.01, ***p < 0.001, ****p < 0.0001.

## Results

### Physicochemical Comparison of the Contrast Agents

**Figure 1:**
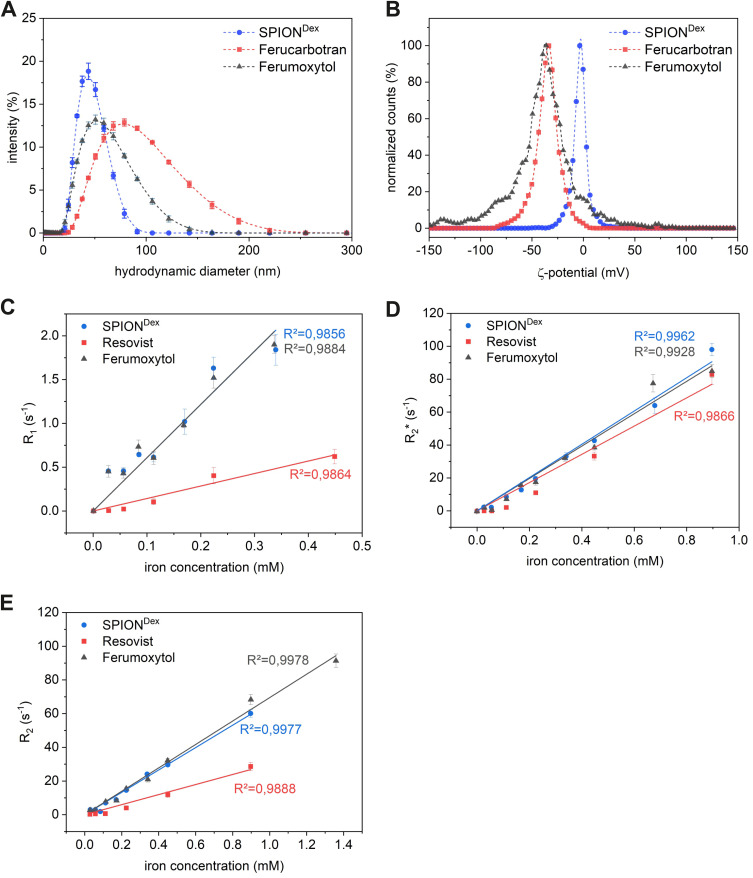
Comparison of physicochemical properties of the particles. (**A**) Intensity-weighted hydrodynamic size distribution. (**B**) $\zeta$-potential distribution at pH 7.4. 3 T MRI derived T_1_ (**C**), T_2_ (**D**) and T_2_* (**E**) relaxation rates as a function of iron concentration. The relaxivity r is derived from the slope of the corresponding linear fit.

**Notes**: For (**A** and **B**): representative result of one experiment. For (**C**–**E**): mean ± SD of n=3 experiments are shown.

**Abbreviations**: R_i_, relaxation rate of T_i_-weighted sequences (i= 1, 2 or 2*), SPION^Dex^, dextran-coated superparamagnetic iron oxide nanoparticles, SD, standard deviation.

In order to better evaluate the differences in the toxicological profile of the different contrast agents and the outcome of the animal experiments, we first determined basic physicochemical parameters, which are summarized in Table 2. With a hydrodynamic size of 35 nm and a low polydispersity index (PDI) of 0.129, SPION^Dex^ were the smallest of the three compounds and had the narrowest size distribution (see Figures 1A, S1a and S1b). Ferumoxytol had a comparable size of 38 nm, whereas ferucarbotran particles were much larger (59 nm). Both ferumoxytol and ferucarbotran possessed a highly negative surface charge (−40 and −35 mV), in contrast to SPION^Dex^ that had only a slightly negative mean zeta potential of −4 mV (Figure 1B). TGA analysis showed comparable organic contents, ie, shell material, of approx 40–45 wt% (Figure S1c–e), but as expected, FTIR revealed the variation in the coating materials (Figure S1f). While all three spectra show typical peaks of carbohydrates, differences are visible in the finger-print region. SPION^Dex^ possessed typical vibrations of crosslinked dextran.^34^ The spectrum of ferucarbotran differs in this respect in the C-O-C vibrational region, and in the appearance of another peak at 1591 cm^−1^, representing the carboxyl functionalization of its dextran shell.^41^ Typical peaks of the polyglucose sorbitol carboxymethyl ether coating were detectable in the spectrum of ferumoxytol.^42^ For 3T MRI, R_1_, R_2_ and R_2_* relaxation rates were measured using different MRI protocols to determine the contrast agent relaxivity values r_1_, r_2_ and r_2_* as a measure of in vitro MRI contrast efficiency (see Figure 1C–E). Ferumoxytol and SPION^Dex^ showed similar relaxivity values, but ferucarbotran was less potent under the tested conditions for all three sequences (see Table 2).

**Table 2:** Summary and Comparison of the Contrast Agent Formulations’ Different Physicochemical Properties

Property	SPION^Dex^	Ferucarbotran	Ferumoxytol
Hydrodynamic size (nm)	35 ± 0.1	59 ± 0.1	38 ± 0.2
Polydispersity index (a.u.)	0.129 ± 0.005	0.180 ± 0.013	0.210 ± 0.001
ζ-Potential (mV) at pH 7.4	−4 ± 0.3	−35 ± 0.4	−40 ± 0.5
Magnetic volume susceptibility normalized to iron concentration (mL/mg)	9.0 × 10^−4^ ± 4 × 10^−6^	7.7 × 10^−3^ ± 9 × 10^−7^	1.5 × 10^−3^ ± 3 × 10^−7^
r_1_ (s^−1^mM^−1^)	6 ± 0.4	1.4 ± 0.1	6 ± 0.4
r_2_ (s^−1^mM^−1^)	66 ± 1.6	30 ± 2.0	67 ± 1.7
r_2_* (s^−1^mM^−1^)	101 ± 3.0	86 ± 5.8	98 ± 4.0

**Note**: Mean ± SD of n=3 experiments are shown.

**Abbreviations**: r_i_, relaxivity of T_i_-weighted sequences (i= 1, 2 or 2*), SPION^Dex^, dextran-coated superparamagnetic iron oxide nanoparticles, SD, standard deviation.

### Nanoparticulate Contrast Agent-Induced Cytotoxicity

**Figure 2:**
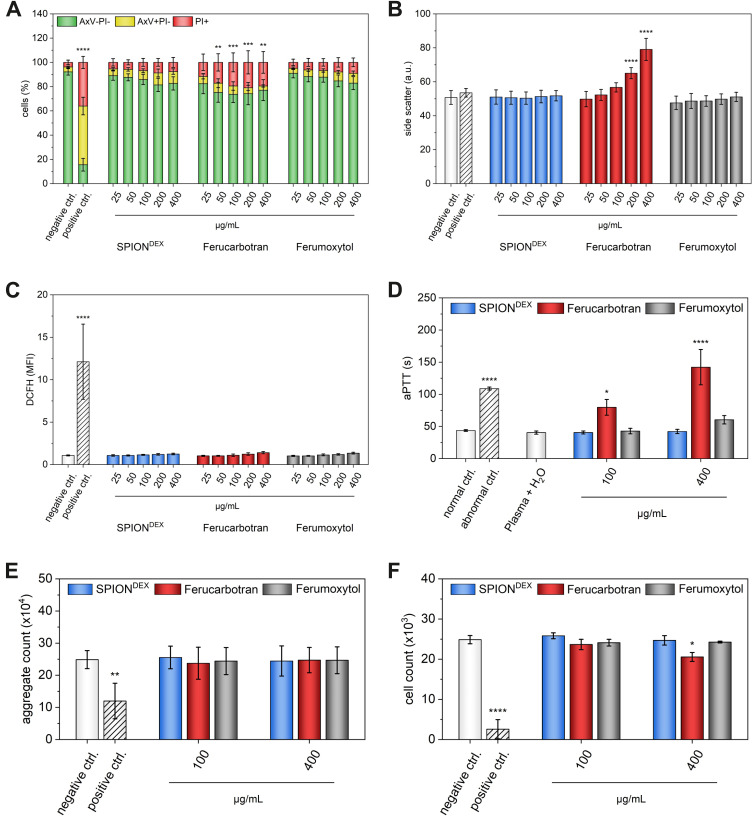
Cytotoxicity of nanoparticles. (**A**) Viability of Jurkat cells after treatment with nanoparticles for 24 h determined by AxV-PI staining. (**B**) Cellular nanoparticle uptake determined by FACS side scatter intensity. (**C**) Reactive oxygen species formation in cells measured by DCFH fluorescence. (**D**) Effect of contrast agents on intrinsic blood coagulation pathway. (**E**) Activation of thrombocytes. (**F**) Formation of neutrophil extracellular traps.

**Notes**: Asterisks mark the significance levels after passed normality test (*p < 0.05, **p < 0.01, ***p < 0.001, ****p < 0.0001). For (**A**–**C**) mean ± SD of n=5 experiments are shown. For (**D**–**F**) mean ± SD of n=3 experiments are shown.

**Abbreviations**: SPION^Dex^, dextran-coated superparamagnetic iron oxide nanoparticles, a.u., arbitrary units, DCFH, 2’-7ʹdichlorofluorescin, aPTT, activated partial thromboplastin time, SD, standard deviation.

In this study, contrast agent cytotoxicity was investigated using the non-adherent human T cell leukemia cell line Jurkat. These cells were treated with iron concentrations up to 400 µg/mL for 24 and 48 h, followed by flow-cytometric examination of cell death markers. Initially, we examined phosphatidylserine exposure by AxV staining, and plasma membrane integrity by PI staining (Figure 2A) in order to distinguish between viable (AxV-PI-), apoptotic (AxV+PI-) and necrotic (PI+) cells. After 24 h incubation with SPION^Dex^ or ferumoxytol, a very weak, dose-dependent impact on cell viability was observed, but it was not statistically significant. In contrast, ferucarbotran significantly reduced cell viability already at 50 µg/mL. The same trend was also evident when measuring the loss of mitochondrial membrane potential (Figure S2a). However, no changes in cell cycle, such as cell cycle block or DNA degradation due to activation of DNAses during apoptosis, were observable upon contrast agent treatment (Figure S2b). Ferucarbotran also showed a significant increase in the side scatter intensity (Figure 2B), indicative of cellular uptake of nanoparticles.^43^ In contrast, side scatter intensity was not increased for SPION^Dex^ and ferumoxytol. To obtain information on the amount of oxygen/nitrogen radicals and cellular glutathione status (oxidized/reduced) after nanoparticle administration, combined staining with 2’,7ʹ-dichlorofluorescin (DCFH) and monobromobimane (MBB) were performed and evaluated using flow cytometry. DCFH staining after 24 h of incubation with the nanoparticles revealed a very weak, yet not significant fluorescence signal increase at the highest tested concentration (400 µg/mL) for ferucarbotran and ferumoxytol (Figure 2C). No significant increase in DCFH signal was observed in cells exposed to SPION^Dex^, even at 400 µg/mL. In addition, the MBB fluorescence was also not significantly altered after 24 h, (Figure S2c) confirming the results of the DCFH measurements.

### Particle Hemocompatibility

To prevent the clogging of blood vessels by agglomerating nanomaterials, their colloidal stability in human blood must be confirmed. To test this, human whole blood was anticoagulated with different substances (lithium heparin, EDTA, sodium citrate) to exclude effects of the anticoagulant itself. Figure S3 shows representative example results from one donor at an iron concentration of 250 µg/mL. Across the all three donors, no particle agglomerates were detectable under the microscope for any of the three nanoparticle formulations. Since the resolution of the used light microscope is about 200 nm, it can be assumed that no agglomerates larger than this limit occur under the tested conditions. In addition, none of the formulations produced hemolysis (Figure S2d). Another important factor is blood coagulation, a complex multicomponent process with three main activation pathways: intrinsic, extrinsic and common. To analyze whether coagulation is induced by the different nanoparticles, platelet-poor plasma was preincubated with the respective nanomaterials for 30 minutes at 37 °C. Figure 2D shows the results of the aPPT test, a measure for the intrinsic blood coagulation pathway. While SPION^Dex^ had no effect on aPPT even at the highest concentration, ferucarbotran massively inhibited the intrinsic pathway and its effect at the highest tested concentration exceeded even the positive control. Ferumoxytol at 400 µg/mL slightly but not significantly increased the aPPT. In addition, the extrinsic pathway determined by the prothrombin time (Figure S2e) was not influenced by the contrast agents, while the common pathway, reflected by the thrombin time values, was significantly inhibited by ferucarbotran at a concentration of 400 µg/mL (Figure S2f). The Interaction of platelets with nanoparticles was investigated using platelet-rich plasma (PRP). After incubation with CA, the aggregation of platelets was determined by flow cytometry. Compared to the negative control, no significant increase in aggregated platelets was determined (Figure 2E). After induction of platelet aggregation in the CA-containing PRP by collagen (10 µg/mL), in the presence of ferucarbotran, lower numbers of platelet aggregates were detected, whereas SPION^Dex^ and ferumoxytol did not interfere with collagen-induced platelet aggregation (data not shown). Beyond these well-known pathways, there is another possibility how SPIONs potentially can induce blood clotting, namely by the formation of neutrophil extracellular traps (NETosis). In order to test this, isolated polymorphonuclear leukocytes (PMN) were incubated with the respective contrast agents for 3 h and the number of not agglomerated PMN was subsequently determined by flow cytometry. The positive control phorbol-12-myristate-13-acetate (PMA) induced NETosis, leading to a reduced amount of free PMN (Figure 2F). In contrast, none of the three formulations affected the PMN count as the positive control but ferucarbotran showed a mild significant reduction at the highest tested concentration.

### Contrast Agents Show Strong Differences in Complement Activation

**Figure 3:**
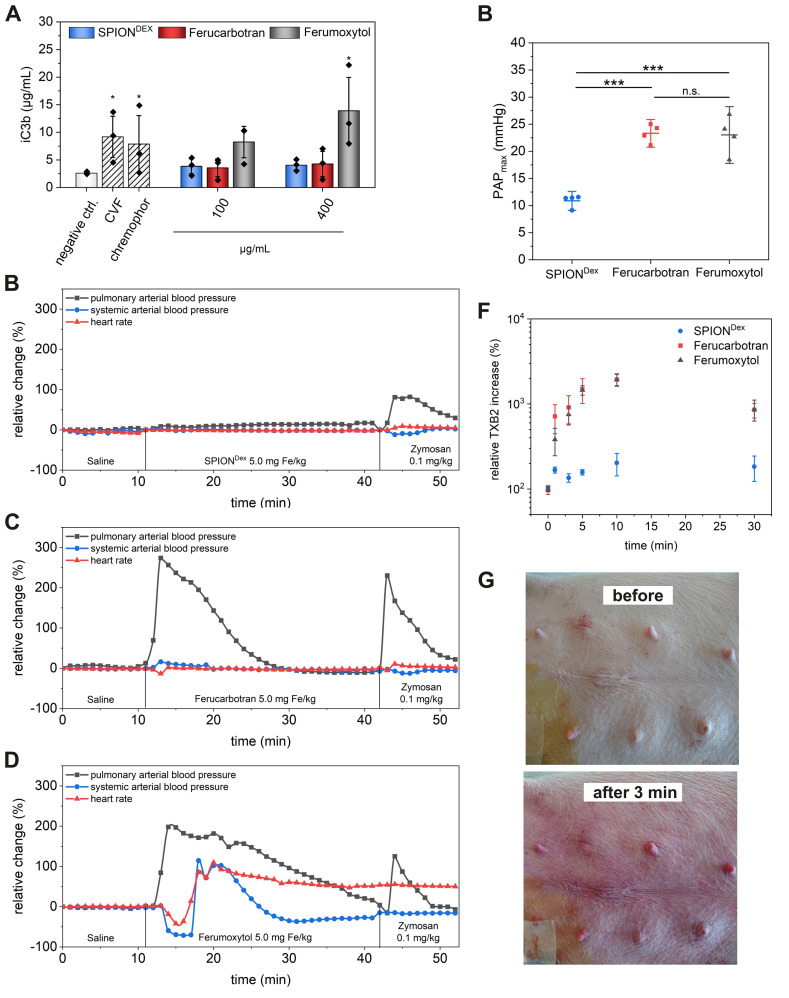
Contrast agent-induced complement activation. (**A**) Determination of the iC3b split product, in human-derived platelet-poor plasma (n=3). Relative hemodynamic changes as indication of in vivo complement activation in a porcine model after administration of SPION^Dex^ (**B**), ferucarbotran (**C**), and ferumoxytol (**D**) at an iron concentration of 5 mg/kg. The latter resulted in anaphylaxis and requiring subsequent cardiopulmonary resuscitation. (**E**) Comparisons of maximum pulmonary artery pressure after contrast agent application (data for both doses, 0.5 and 5.0 mg Fe/kg, are pooled). (**F**) Normalized thromboxane B2 levels in pigs acquired from blood samples collected during application. (**G**) Example image of flushes appearing in the abdominal region after ferumoxytol application.

**Notes**: Asterisks mark the significance levels after passed normality test (***p < 0.01, ***p < 0.001, ****p < 0.0001). For (**A**): mean ± SD of n=3 experiments are shown. For (**B**–**D**): representative result of one experiment are shown. For (**E** and **F**): mean ± SD of n=4 experiments are shown (data for both doses, 0.5 and 5.0 mg Fe/kg, are pooled).

**Abbreviations**: SPION^Dex^, dextran-coated superparamagnetic iron oxide nanoparticles, iC3b, protein fragment that is part of the complement system, CVF, cobra venom factor, PAP_max_, maximum pulmonary artery pressure, TXB2, thromboxane B2, SD, standard deviation.

The complement system is an essential part of the innate immunity, contributing to a non-specific host defense. It consists of a set of plasma proteins that activate each other by cleavage. The complement cascade can be activated by immune complexes (classical pathway), in antibody-independent manner (alternative pathway), or by mannose-binding lectin (lectin pathway). Here, the formation of the cleavage product iC3b that occurs in all three pathways was analyzed using ELISA, showing a strong signal for the control substance cobra venom factor (CVF), but also for Cremophor-EL, which is used in pharmaceutical industry as an excipient to dissolve hydrophobic drugs, eg, Taxol, and is known to form nanosized micelles causing complement activation.^44^ Ferumoxytol strongly induced iC3b formation and the extent of this increase exceeded even the effect of the Cremophor-EL mix (Figure 3A). In samples treated with ferucarbotran or SPION^Dex^ no significant iC3b increase was detected. The data also demonstrated large variability in the responses among different donors. The next step was to evaluate the complement activation-related pseudoallergy (CARPA) response in a porcine model.^33^,^45^ Prior to the injection of the respective contrast agent at 5 mg iron per kg body weight, saline was injected as negative control. Each experiment was then finished by the injection of zymosan as positive control. The comparison of hemodynamic changes induced by the three nanoparticle formulations demonstrated significant differences in animal responses to injections. Administration of SPION^Dex^ (Figure 3B) showed only a very mild increase in PAP (approx 15%) and no changes in heart rate (HR) or systemic arterial blood pressure (SAP) were observed. In contrast, ferucarbotran (Figure 3C) evoked a massive increase in PAP. Ferumoxytol administration (Figure 3D) did not only induce a strong PAP increase but also led to anaphylaxis that required subsequent cardiopulmonary resuscitation (CPR) involving heart massage and intra-cardiac epinephrine injection, resulting in an increase in HR and SAP. Animals treated with particles at a dose reduced to only one-tenth of the original particle dose (ie, to 0.5 mg iron per kg body weight) showed the same behavior (Figure S4a–c). Besides the significant increase in maximal PAP for all ferucarbotran- and ferumoxytol-treated animals (Figure 3E), an increase in the biomarker TXB2 (Figure 3F), as well as a decrease in white blood cells (Figure S4d) and platelet counts (Figure S4e) was observable, too. Beyond that, rashes and flushes appeared in some of these animals shortly after the injection (Figure 3G).

### Kinetics of Liver Imaging in Rats

**Figure 4:**
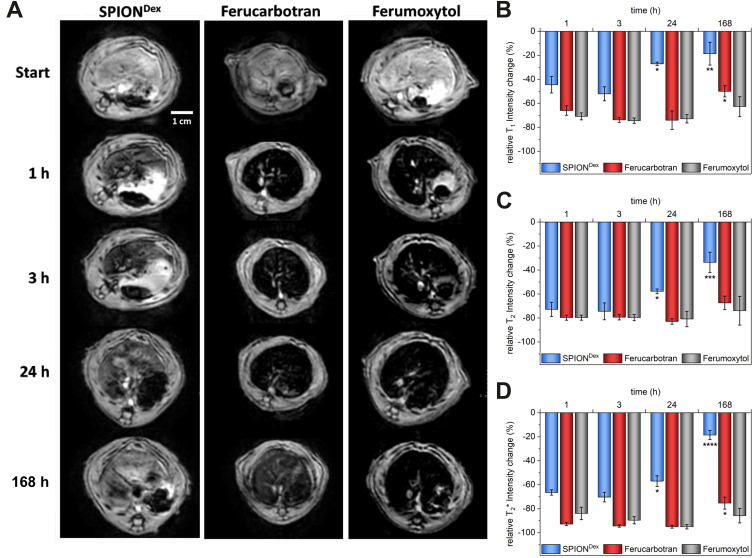
MRI experiments in rats. (**A**) Exemplary transversal T_2_*-weighted MRI of a rat’s liver before and at specific time points after administration of contrast agents at an iron dose of 2.6 mg Fe /kg. Qualitative evaluation of liver uptake and elimination of contrast agents from the liver based on T_1_-weighted (**B**), T_2_-weighted (**C**), and T_2_*-weighted (**D**) scans (n=3).

**Notes**: Asterisks mark the significance levels after passed normality test (*p < 0.05, **p < 0.01, ***p < 0.001, ****p < 0.0001). Mean ± SD of n=3 experiments are shown.

**Abbreviations**: SPION^Dex^, dextran-coated superparamagnetic iron oxide nanoparticles, SD, standard deviation.

The comparison of liver imaging performance and kinetics was done in rats. Accordingly, T_1_- (Figure S5a), T_2_- (Figure S5b) and T_2_*-weighted MRI scans (Figure 4A) of each animal were performed before and at 1 h, 3 h, 24 h and 168 h after particle injection at a dose of 2.6 mg Fe/kg. All three MRI sequences showed strong iron oxide-induced signal attenuation as early as 1 h after contrast agent administration, regardless of the contrast agent used. However, the time course of clearance strongly differed for the different particle types. While the signal attenuation in T_2_*-weighted images induced by SPION^Dex^ became significantly smaller beyond 24 hours, it remained strongly visible even after one-week post administration in animals treated with ferucarbotran or ferumoxytol. Since the interpretation of MRI images is often highly subjective in nature, several areas were manually selected on the liver parenchyma during each scan to compare the normalized mean signal intensity of these areas with the pre-contrast scans. Care was taken to avoid portal veins, hepatic veins, or imaging artifacts when selecting areas. The results shown in Figure 4B–D confirmed that while all three candidates produce a strong negative contrast already after 1 h, especially in the T_2_- and T_2_*-weighted MRI images relevant for iron oxide-based contrast agents, a strong recovery of the signal intensity to the pre-administration status within 1 week was only observable for SPION^Dex^.

## Discussion

Iron oxide-based contrast agents represent a viable alternative to GBCA, at least in some areas of applications. The aim of the current study was to evaluate the risk-to-benefit profile of different formulations and the potential advantages of SPION^Dex^ over ferucarbotran and ferumoxytol, which previously showed adverse effects in humans. In order to better understand the differences observed among the contrast agents, their basic physicochemical properties were compared. While the coating of SPION^Dex^ consists of cross-linked dextran, the FTIR spectrum of ferucarbotran showed a less smeared C-O-C vibrational region, indicating the absence of cross-linking, and an appearance of another peak at 1591 cm^−1^, representing the carboxyl functionalization of its dextran shell, that contributes to its high surface charge.^41^ Also in ferumoxytol, carboxyl groups are responsible for the high negative surface charge.^42^ Despite strong differences in surface charge, all three nanoparticle formulations were characterized with a good colloidal stability in aqueous media and blood, which can be attributed to good electrosteric or steric stabilization due to their respective carbohydrate coating.^46^ Despite its larger hydrodynamic size as compared with the other two formulations, ferucarbotran has lower relaxivity values in the direct comparison at the given parameters. However, relaxivity values are highly dependent on the used MRI parameters, as an r_2_ value of 151 mM^−1^s^−1^ was previously reported for ferucarbotran.^29^ Typical r_1_ and r_2_ values for GBCAs are both in the range of approx 5 s^−1^mM^−1^.^47^ Ferumoxytol and SPION^Dex^ therefore have 13 times higher r_2_/r_1_ and 20 times higher r_2_*/r_1_ ratio while the respective ratios are 6 and 17 times higher for ferucarbotran. This explains the ability of iron-oxide-based nanoparticles to create negative contrast at much lower concentrations as required for GBCAs to create positive contrast. Interestingly, the differences in relaxivities among the investigated iron oxide-based contrast agent do not translate directly into the in vivo imaging performance in rats. A simple explanation may be that in the in vitro experiments water is used instead of, eg, serum, which can have an effect on the relaxivity.^48^ Further, the biodistribution behavior of the contrast agents may differ. SPION^Dex^ may have a longer blood half-life, so that their tissue/blood pool level differs from that of ferumoxytol or ferucarbotran. Interestingly, the MRI signal recovery in the rats’ livers implies that SPION^Dex^ are metabolized or excreted from the liver more rapidly than other formulations, whereas ferucarbotran and ferumoxytol remain much longer in the liver and thus presumably in the patient’s body. As a result, SPION^Dex^ administration could enable the repeated imaging for disease monitoring.

For the purpose of serial imaging, however, the contrast agent must be free of the risk of hypersensitivity reactions upon repeated administration, which may affect cellular, hemostasis-related and immune processes. Among the tested contrast agents, only ferucarbotran showed a significant cytotoxic effect in Jurkat cells, which correlated with an increased side scatter intensity, a measure of cellular particle uptake.^43^ In contrast, side scatter intensity was not affected by treatment with SPION^Dex^ or ferumoxytol, suggesting only a limited – if any – internalization of these particles. In previous studies, SPION^Dex^ showed no cytotoxic effects on human endothelial cells, macrophages and THP-1 cells.^34^ At the highest tested concentrations of ferumoxytol and ferucarbotran, DCFH and MBB measurement indicated a small, but not statistically significant amount of reactive oxygen species (ROS). As a result, no upregulation of the thiol levels was observed in the treated cells, which may indicate that the induced oxidative stress was not strong enough to disturb redox-homeostasis of the cells.

Interference with blood coagulation pathways is one of the potential pitfalls in the clinical translation of nanoparticles.^49^ Our results demonstrated that ferucarbotran strongly inhibited the intrinsic pathway of coagulation cascade, to the degree that exceeded the abnormal plasma control at the highest particle concentration. Ferucarbotran-induced inhibition of the common pathway was weaker, but still present. Incubation with ferumoxytol also caused an inhibition of the intrinsic pathway, but its effect was far less pronounced and SPION^Dex^ did not influence any of the coagulation pathways under the given conditions. Neither the extrinsic pathway nor platelet activation was affected by any of the contrast agents.

The most important aspect in clinical translation of parenterals is immunogenicity, especially when those agents are used for diagnostic purposes. In treatment and prevention, eg vaccination, a specific immune response is desired, but in diagnostics such a response usually leads to adverse effects, therefore stringent investigations of particle immunogenicity are required.^50^ Upon intravascular injection of particles, the first line of immune defense they encounter are neutrophils, which either remove them by phagocytosis, inactivate them, or immobilize them by formation of NETs. As previously reported, nanoparticles can induce NET formation depending on their physicochemical properties such as size, coating and agglomeration status,^51^ but our data indicated that only ferucarbotran at 400 µg/mL led to a moderate increase in NET formation. The next line in the immune defense, the complement system, is an essential part of the innate immunity, contributing to a non-specific host defense and can be activated by immune complexes (classical pathway), by an antibody-independent manner (alternative pathway), or by mannose-binding lectin (lectin pathway). The complement system can support recognition and clearance of pathogens and/or particles by their opsonization (C3b), but the uncontrolled production of proinflammatory anaphylatoxins (C3a, C4a, C5a) upon complement activation can stimulate the release of additional inflammatory mediators (eg histamine) by immune cells. This potentially life-threatening cascade of inflammatory events can be observed upon particle injection in vivo, in a model of CARPA.^45^ Several nanoparticulate drugs in clinical use (eg Doxil, Taxol) have been reported to cause hypersensitivity reactions similar to CARPA in patients.^52^ In vitro complement activation by nanoparticles in plasma have been reported to correlate with in vivo complement-mediated reactions.^53^,^54^ To gain understanding of the contrast agents’ potential to activate the complement system, we first incubated them in vitro with human sera. As expected, there was a large variability in the responses among donors, because complement is part of the innate immune system. However, a very clear tendency towards activation of the complement system was observed in ferumoxytol-treated samples, partially exceeding the effects of the positive controls. Subsequently, we tested whether the contrast agents cause CARPA reactions in an in vivo porcine model.^39^ Symptoms of CARPA include hemodynamic and cardiopulmonary changes, with the increase in pulmonary arterial pressure (PAP) in pigs being the most common.^45^ In addition, hematologic changes such as leukopenia or leukocytosis, thrombocytopenia, skin reactions and elevated thromboxane B2 (TXB2), as well as C-reactive protein levels are also important biomarkers of CARPA.^45^ The results demonstrated that both ferumoxytol and ferucarbotran induced strong CARPA reactions in pigs even at low doses. Of note, the occurrence of CARPA in response to ferumoxytol was proportional to the increase of complement split product induced by this formulation in vitro. In contrast, the CARPA-genic activity of ferucarbotran did not correlate with its in vitro effects. This may be due to the fact that multiple pathways are activated during CARPA in vivo, including anaphylatoxin induction of mast cells and macrophages, as well as subsequent release of vasoactive and inflammatory mediators, in addition to C-activation. Nevertheless, the CARPA reactions observed in ferumoxytol-treated animals were more severe and the resulting anaphylaxis required subsequent cardiopulmonary reanimation. This is a very important finding as CARPA reactions are very likely responsible for the occurrence of adverse/hypersensitivity effects in patients administered SPION-based contrast agents and may affect risk-to-benefit profile of these formulations.^29^,^30^,^54^,^55^ Irrespective of the applied dose, SPION^Dex^ did not evoke any CARPA reactions. The reason for this could be related to the design and morphology of these nanoparticles, in which they differ from both ferucarbotran and ferumoxytol. Due to the crosslinking of dextran coating in SPION^Dex^, the sugar structure of the coating is altered. In contrast to its native form, such a crosslinked dextran structure actually prevents complement activation in mice, as shown by Wang et al.^56^ In addition, according to Chao et al, an exposed iron oxide surface provides an anionic charge pattern recognizable by scavenging receptors.^57^,^58^ It can thus be assumed that crosslinking of dextran adsorbed on the surface of the particles leads to a denser surface coverage and, therefore, to a better shielding against these receptors.

## Conclusion

In all toxicological studies, the SPION^Dex^ performed equally or significantly better than the two reference products. As a result, upon their clinical translation, SPION^Dex^ may provide a safer diagnostic agent with less adverse side effects. SPION^Dex^ are already upscaled and the cGMP compliant production processes required for translation are well advanced. Before they can be ultimately applied in their first-in-human trials, they have to pass mandatory regulatory safety tests performed by certified laboratories.
